# Supplementary material for: Nanotopography reveals metabolites that maintain the immunomodulatory phenotype of mesenchymal stromal cells
Source: Nat Commun. 2023 Feb 10;14:753. doi: 10.1038/s41467-023-36293-7 (PMC9918539; doi:10.1038/s41467-023-36293-7)
Supplement: Supplementary file 1 — Supplementary Information [file 41467_2023_36293_MOESM1_ESM.pdf]

Supplementary Information

Nanotopography reveals metabolites that maintain the immunomodulatory phenotype of mesenchymal stromal cells

Ewan A Ross, Lesley-Anne Turner, Hannah Donnelly, Anwer Saeed, Monica P. Tsimbouri, Karl V Burgess, Gavin Blackburn, Vineetha Jayawarna, Yingbo Xiao, Mariana AG Oliva, Jennifer Willis, Jaspreet Bansal, Paul Reynolds, Julia A Wells, Joanne Mountford, Massimo Vassalli, Nikolaj Gadegaard, Richard OC Oreffo, Manuel Salmeron-Sanchez and Matthew J Dalby.

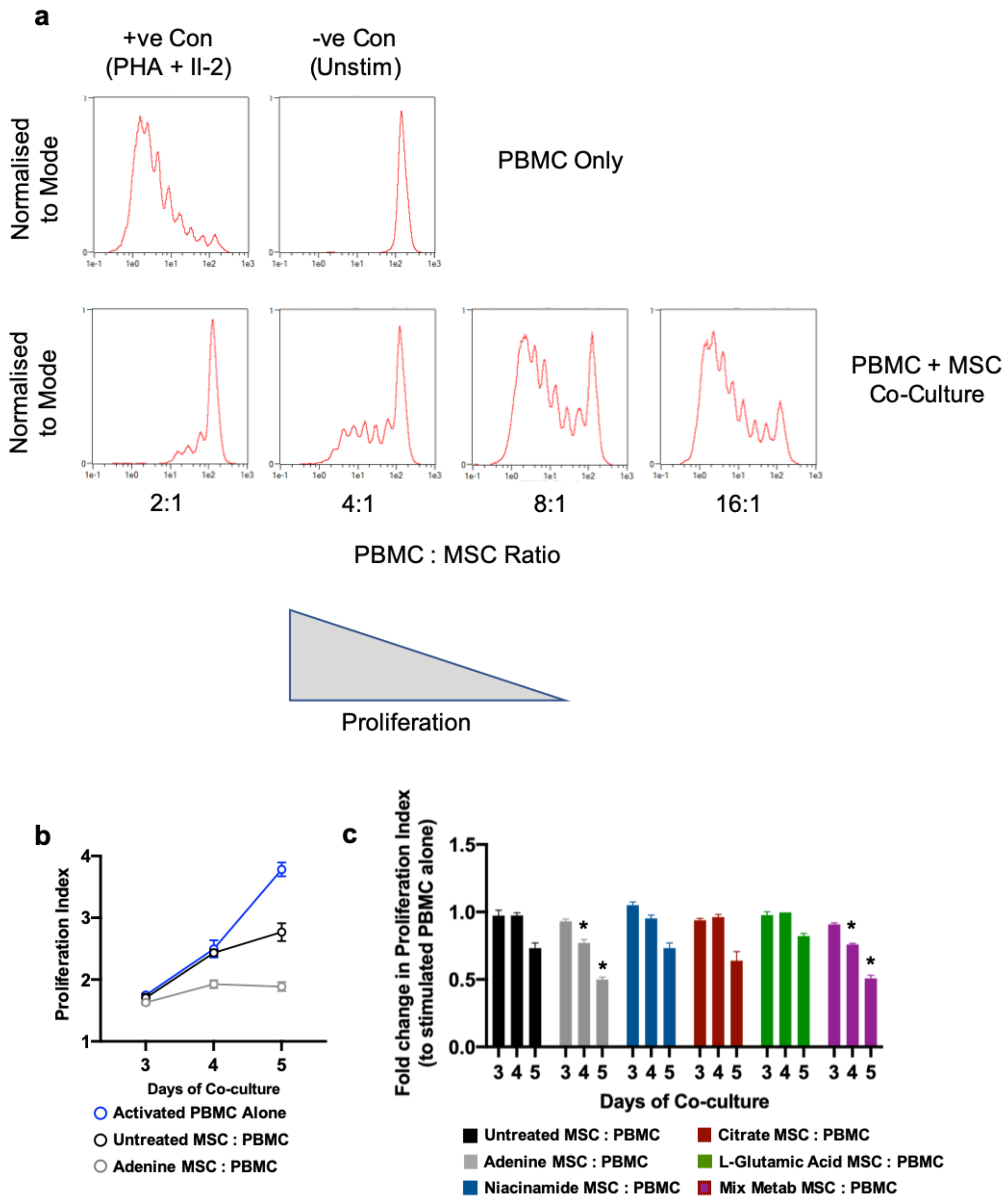

Fig. S1. CFSE immunosuppression assay. (a) PBMCs were isolated from peripheral blood, labelled with CFSE, and stimulated to proliferate with 5µg/ml PHA-P and 100U/ml IL-2. PBMCs were then added to MSCs at defined ratios and co-cultured for 5 days. CFSE dilution was assessed by flow cytometry. Positive control was stimulated PBMCs on their own; negative control was CFSE-labelled PBMCs in the absence of PHA-P and IL-2. (b) Timecourse of the proliferation index of PBMCs in co-culture with MSCs. (c) Timecourse of the fold change in the proliferation index (compared to stimulated PBMCs alone) in co-culture with MSCs treated with metabolites for 14 days, Graphs in b and c show mean ± S.D (n=4 replicates per group from one donor. Comparisons are of the increased effects of metabolite treatment to the untreated MSCs alone in T cell suppression by two-way ANOVA with Dunnett's multiple comparison test; p values, at day 4 adenine p=0.0016 and mixed metabolites p=0.0017, at day 5 adenine p=0.0079 and mixed metabolite p=0.0066). Representative of two independent experiments. Source data are provided as a Source Data file.

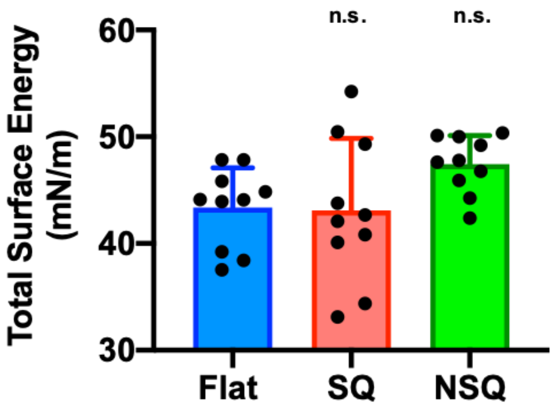

Fig. S2. Total surface energy for different topographical surfaces measured using OWRK method. The results suggest that different nanotopographical surfaces do not involve changes in surface energy. Graphs show mean ± S.D. of n=10 measurements per group, comparisons by one way ANOVA with Dunnetts's test of multiple comparisons. Source data are provided as a Source Data file.

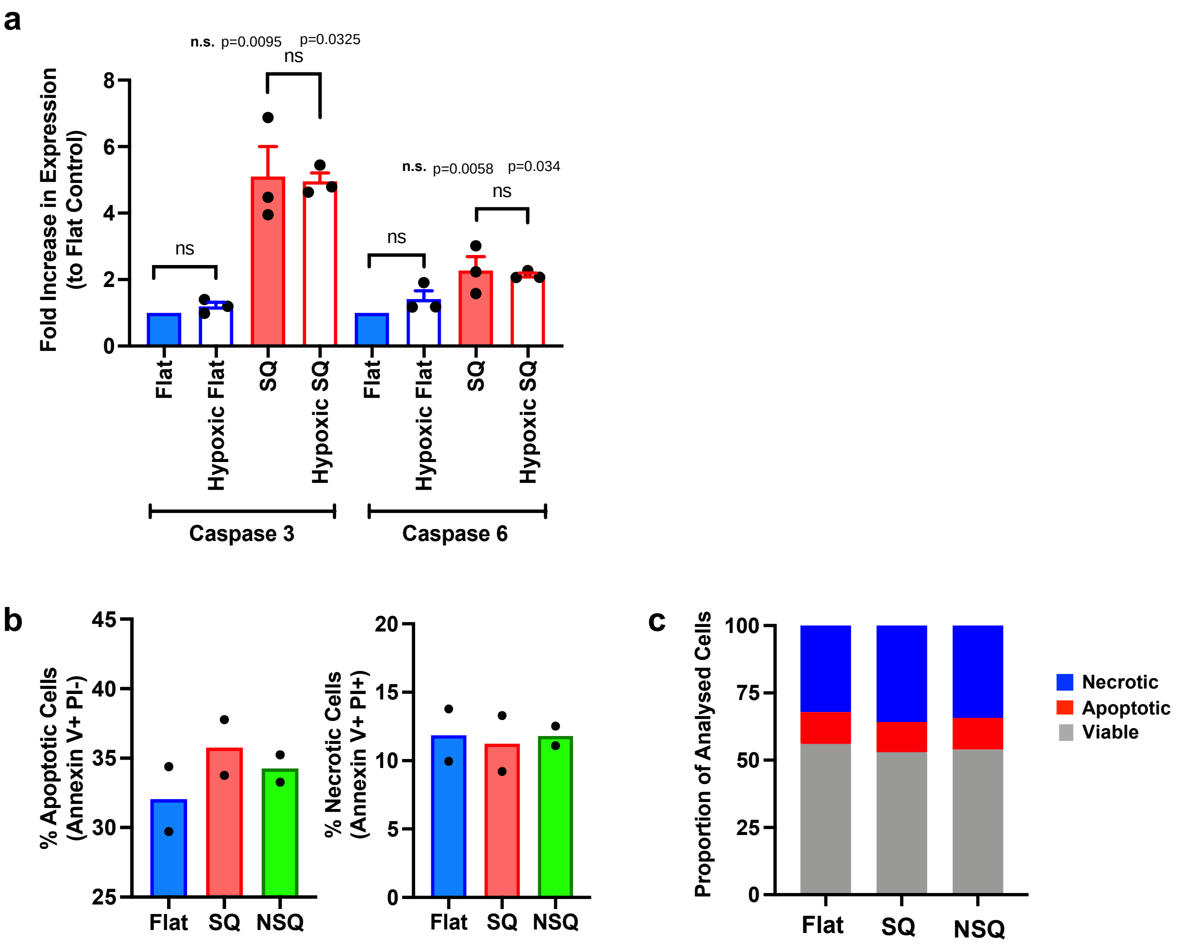

Fig. S3. Effects of topographies on MSC susceptibility to apoptosis. (**a**) MSCs culture on topographies in normoxic or hypoxic environments or with the ROCK inhibitor Y27632 and activation of Caspase 3 and 6 quantified using immunofluorescent staining by in cell western (n=3 donors, mean ± SEM; Mann-Whitney paired analysis in brackets; 2-way ANOVA with Dunnetts multiple comparison test above). **(b)** MSCs were cultured with activated PBMCS at a 1:10 ratio for 4 hours, and the induction of apoptosis assessed by Annexin-V and PI staining by flow cytometry (n=3 replicates per group; mean ± S.D.; n=2 independent donors). The three populations identified (Viable, Annexin-V^-PI^-; Apoptotic, Annexin-V^+PI^-; Nectrotic, Annexin-V^+PI^+) for one patient represented as a stacked graph (**c**). Source data are provided as a Source Data file.

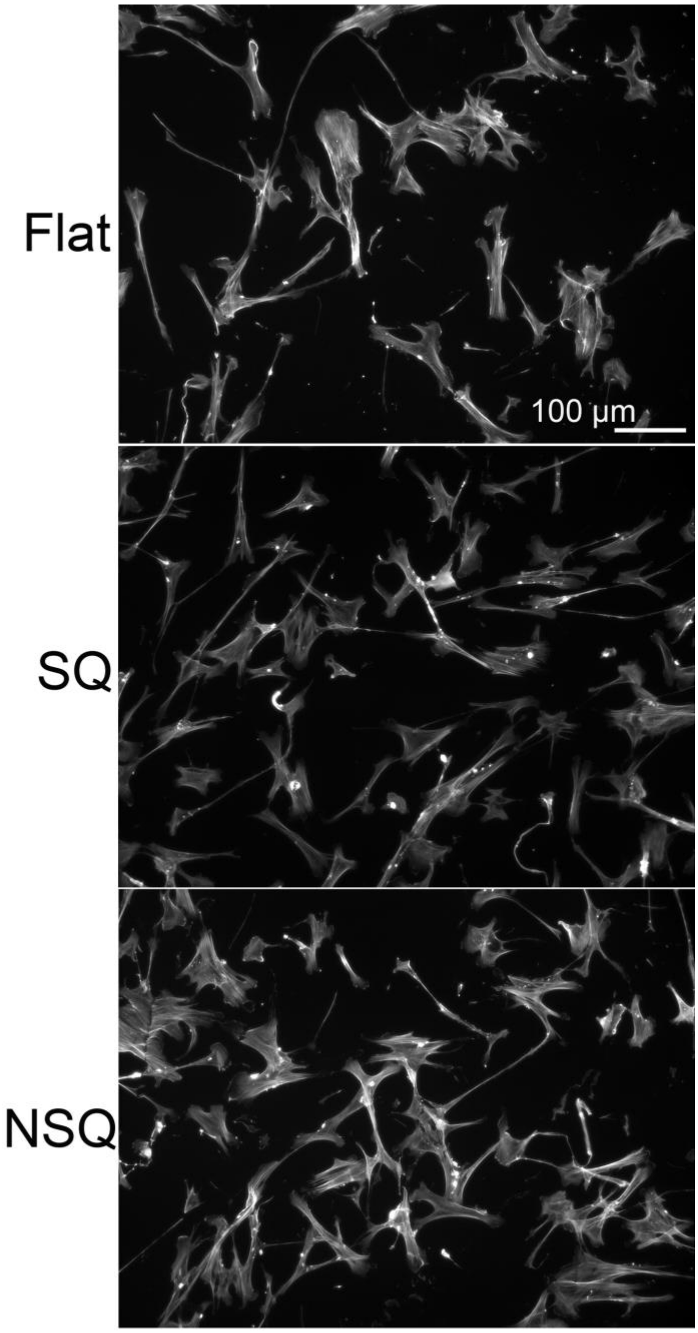

Fig. S4. Actin cytoskeleton staining. Actin staining after 3 days of culture on topographies revealing increased actin stress fibre organisation for MSCs on NSQ compared to MSCs on SQ. Representative images shown are taken from one donor cultured on two separate topography types. Images are representative of four individual donors cultured on topographies.

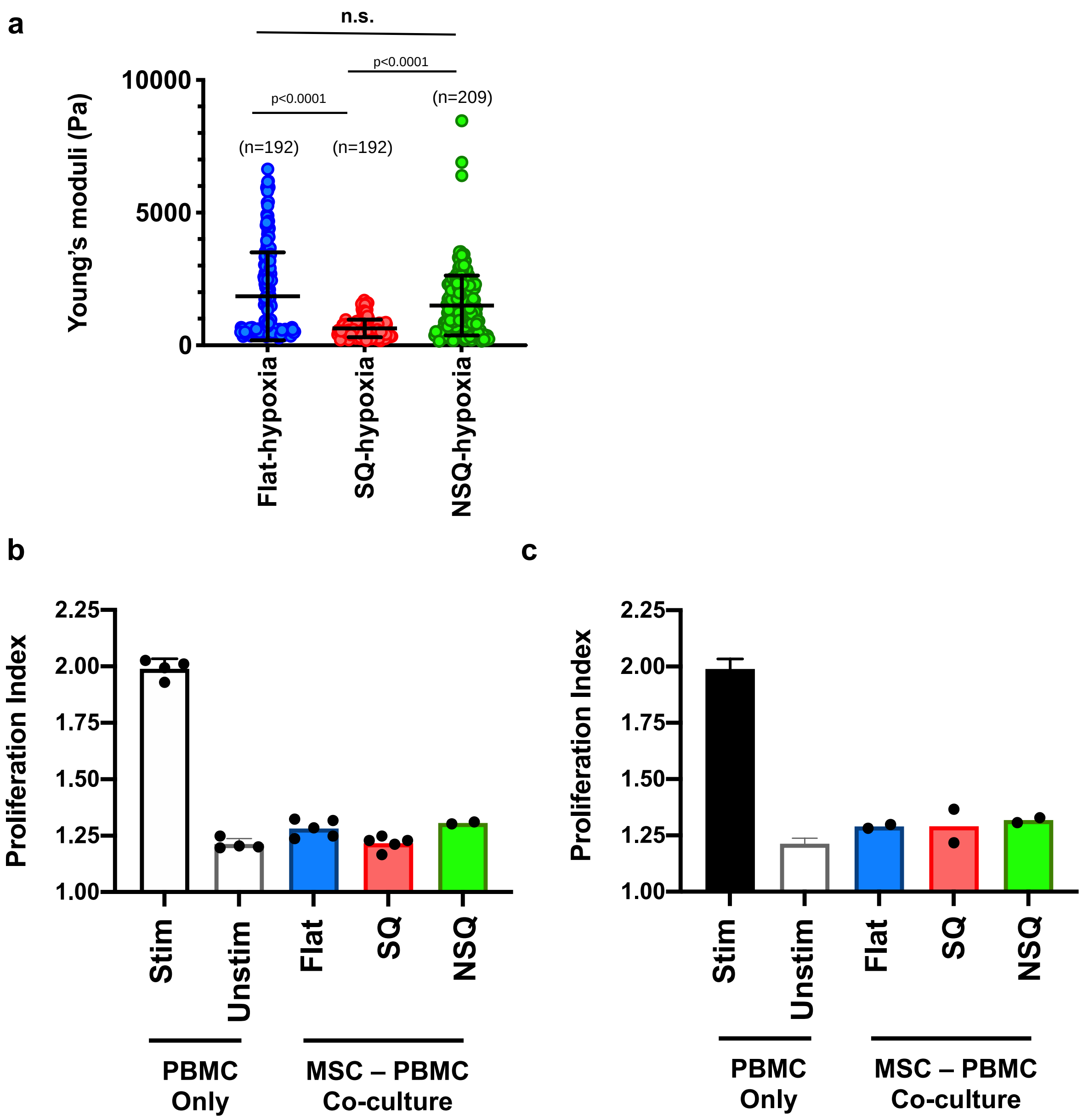

Fig. S5. Changes in cell membrane stiffness of MSCs cultured on nanotopographies in hypoxic conditions. (a) MSCs were cultured for 7 days on topographies in a hypoxic chamber (1% oxygen tension). Changes to cell membrane stiffness were assessed using nanoindentation. Numbers in brackets represent the number of individual measurements. Graph shows mean ± S.D., comparisons by one way ANOVA with Kruskal-Wallis test of multiple comparisons. (b) Stro-1^{+} MSCs were cultured on nanotopographies for 14 days in hypoxia, then co-cultured with CFSE-labelled, PHA and IL-2 stimulated PBMCs for a further 5 days. CFSE dilution was quantified by flow cytometry. Graph in (b) shows representative results from one co-culture (n=2-5 topographies per group, mean ± S.D.). Graph in (c) shows mean proliferation index of 2 donors (each point is the mean of n=2-5 topographies per donor per condition). Source data are provided as a Source Data file.

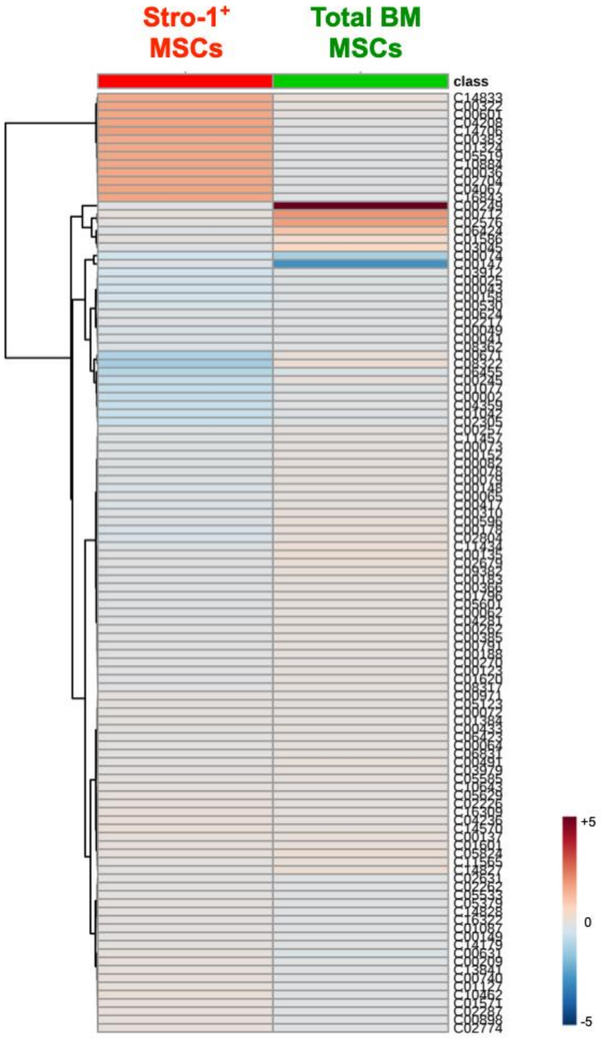

Fig. S6. Changes in metabolic profiles of MSCs on topographies. Stro-1^{+} (red) or total BM (green) MSCs were cultured on SQ or flat surfaces for 7 days, and the metabolic profile of MSCs was determined by analysis using IPA software. Heatmap is the mean of 6 individual topographies.

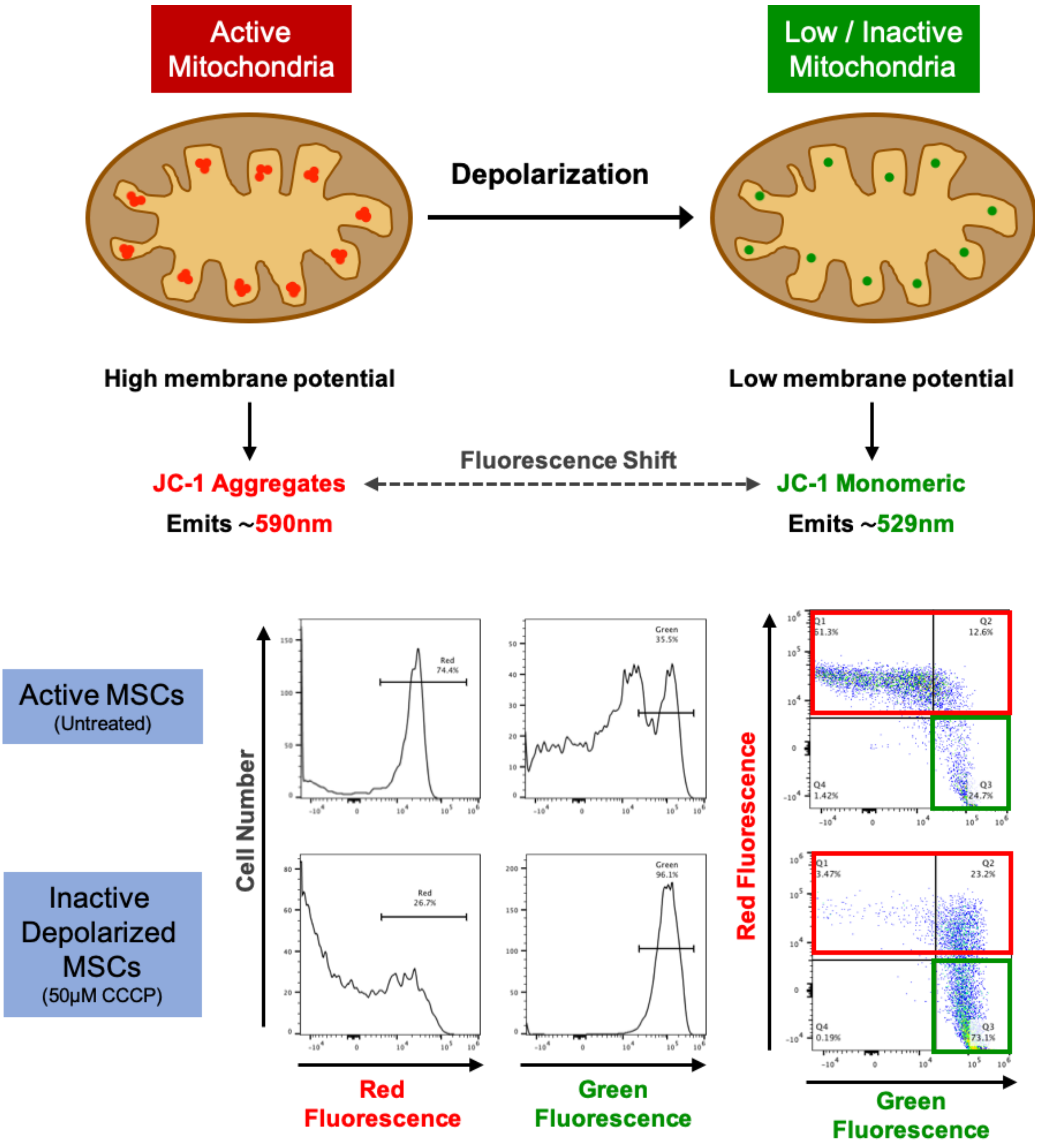

Fig. S7. Mitochondrial activity measured using JC-1. MSCs were labelled with 2 µM JC-1 for 30mins before being detached with trypsin and their fluorescence quantified by flow cytometry. Active mitochondria accumulate red fluorescent dye, whereas depolarised, inactive mitochondria remain green. Depolarisation can be induced by treating MSCs with 50 µM of the protonophore CCCP. Quantification of red and green fluorescence by flow cytometry provides a functional readout of mitochondrial activity. The JC-1 ratio allows mitochondrial function to be compared between individual experiments and donors.

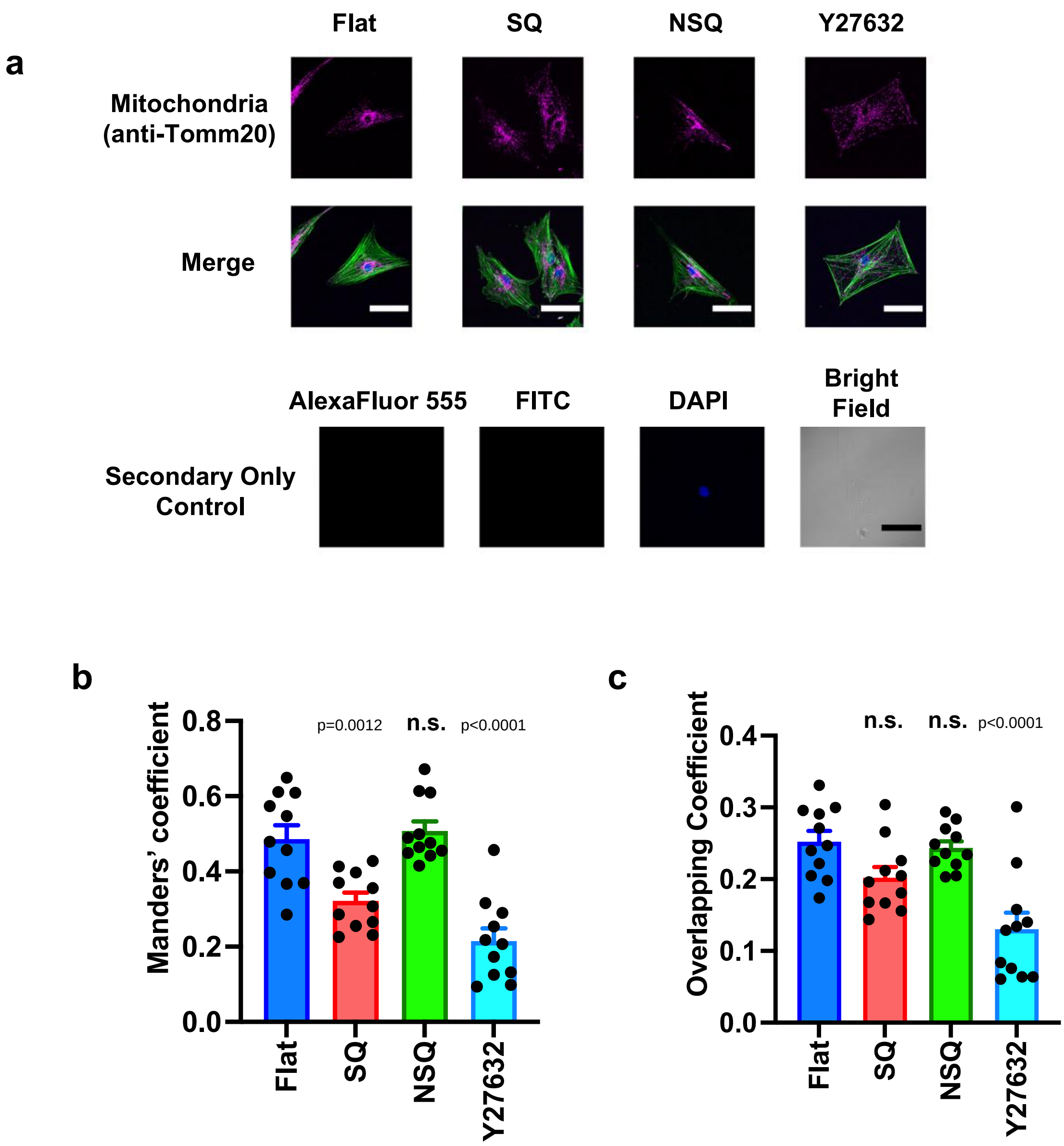

Fig. S8. Effects of nanotopography on mitochondrial distribution in MSCs. (a) Cells were grown on topographies for 7 days before fixation and immunofluorescent staining for total mitochondria (anti-Tomm20 antibody; purple) and phalloidin-FITC to label actin. Super resolution microscopy was performed and the co-localisation of mitochondria with actin filaments was evaluated using image analysis (b and c). Graphs show the mean and S.E.M of where each point represents an independent field of view analysed. Statistical comparisons by two way ANOVA with Dunnett's test of multiple comparisons. Source data are provided as a Source Data file.

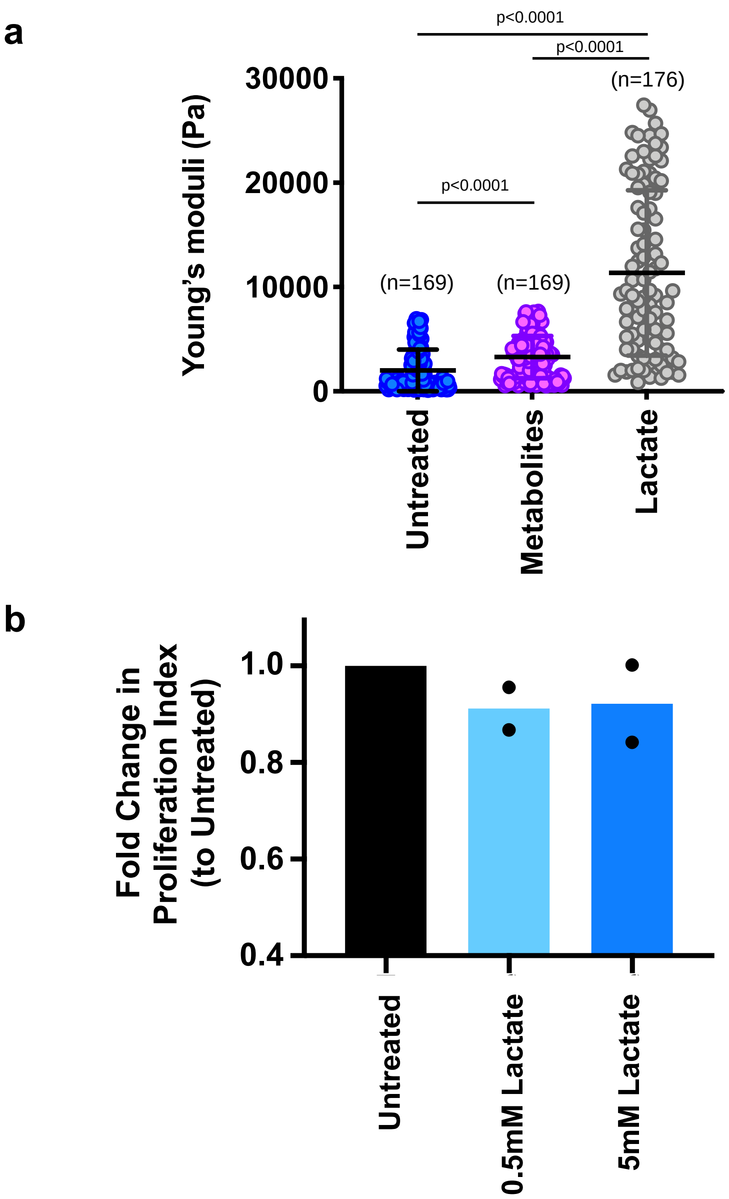

Fig. S9. Effects of exogenous lactate on MSC physiology. (a) MSCs were cultured for 7 days in the presence or absence of 5mM lactate. Changes to cell membrane stiffness were assessed using nanoindentation. Numbers in brackets represent the number of individual measurements**.** Graph shows mean ± S.D., comparisons by one way ANOVA with Kruskal-Wallis test of multiple comparisons. (b) MSCs were cultured for 7 days with 0.5mM or 5mM lactate before co-culturing with CFSE-labelled, PHA and IL-2 stimulated PBMCs for a further 5 days. CFSE dilution was quantified by flow cytometry**.** Graph shows mean of two independent donors (each point is the mean of n=4 replicates per donor). Source data are provided as a Source Data file.

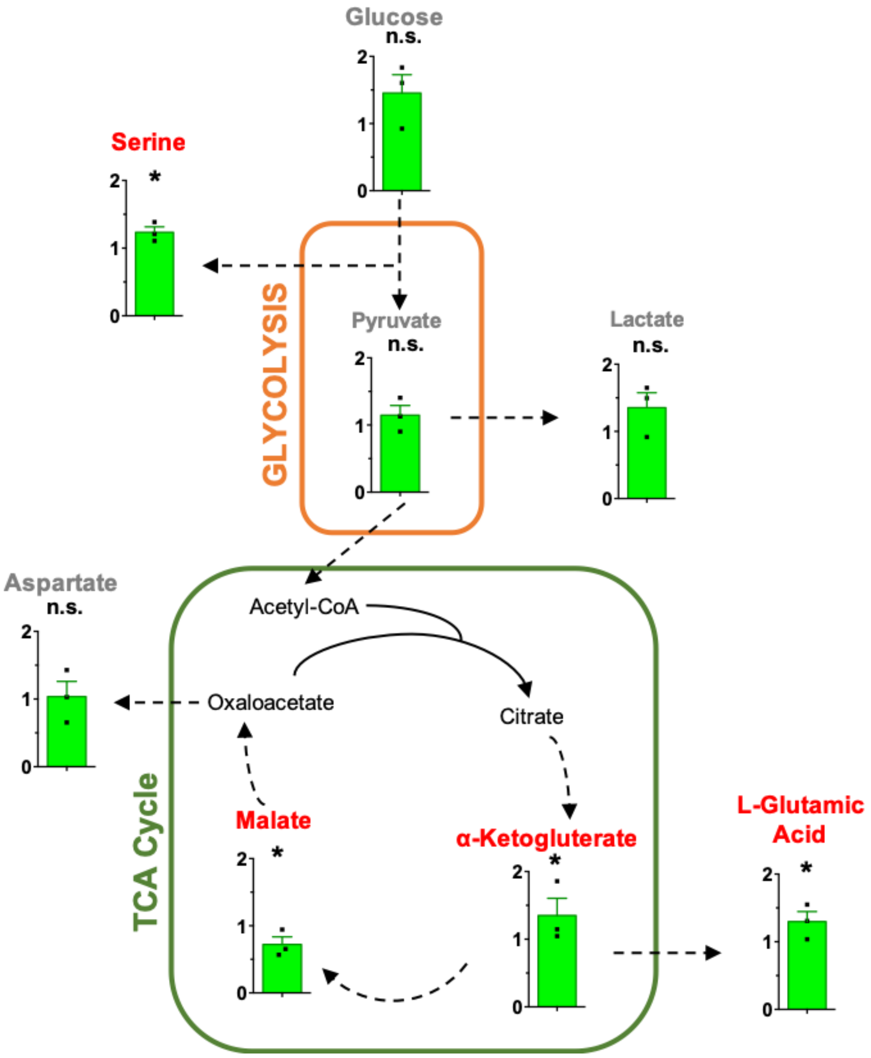

Fig. S10. Metabolic tracing of [$^{13}C_6$]-glucose in MSCs cultured on NSQ nanotopography. Stro-1$^+$ MSCs were cultured on NSQ or flat nanotopographies for 11 days, followed by a further 3 days in the presence of [$^{13}C_6$]-glucose. LC-MS was used to measure the conversion and abundance of [$^{13}C_6$]-labelled metabolites. Graphs show the fold change in [$^{13}C_6$]-labelled metabolites in MSCs cultured on NSQ relative to flat nanotopographies. The results show increase in mitochondrial respiration as indicated by increased ^{13}C incorporation in ketoglutarate and malate. (n=3 independent experiments; each point is the mean of 4 topographies per group; mean ± SEM). Direct comparisons by two-tailed student T-test (Mann-Whitney), *$p < 0.05$; n.s., non-significant). Source data are provided as a Source Data file.

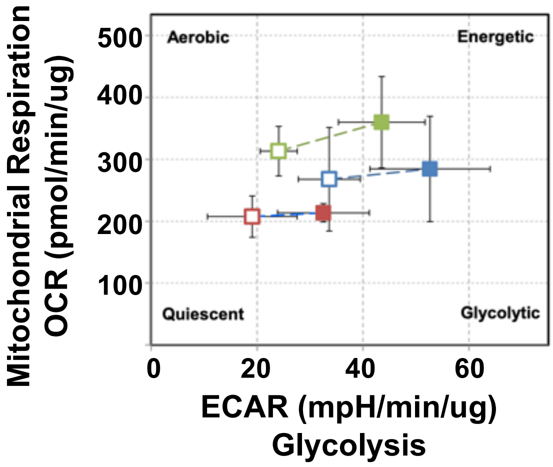

Fig. S11. Seahorse energy map of metabolite treated Stro-1$^+$ MSCs. Generated by plotting extracellular acidification rate (ECAR) versus oxygen consumption rate (OCR) values. Untreated cells in red, Adenine in blue and Niacinamide treated cells in green. Data from mean ± SEM of n=3 technical replicates from n=3 independent donors. Source data are provided as a Source Data file.

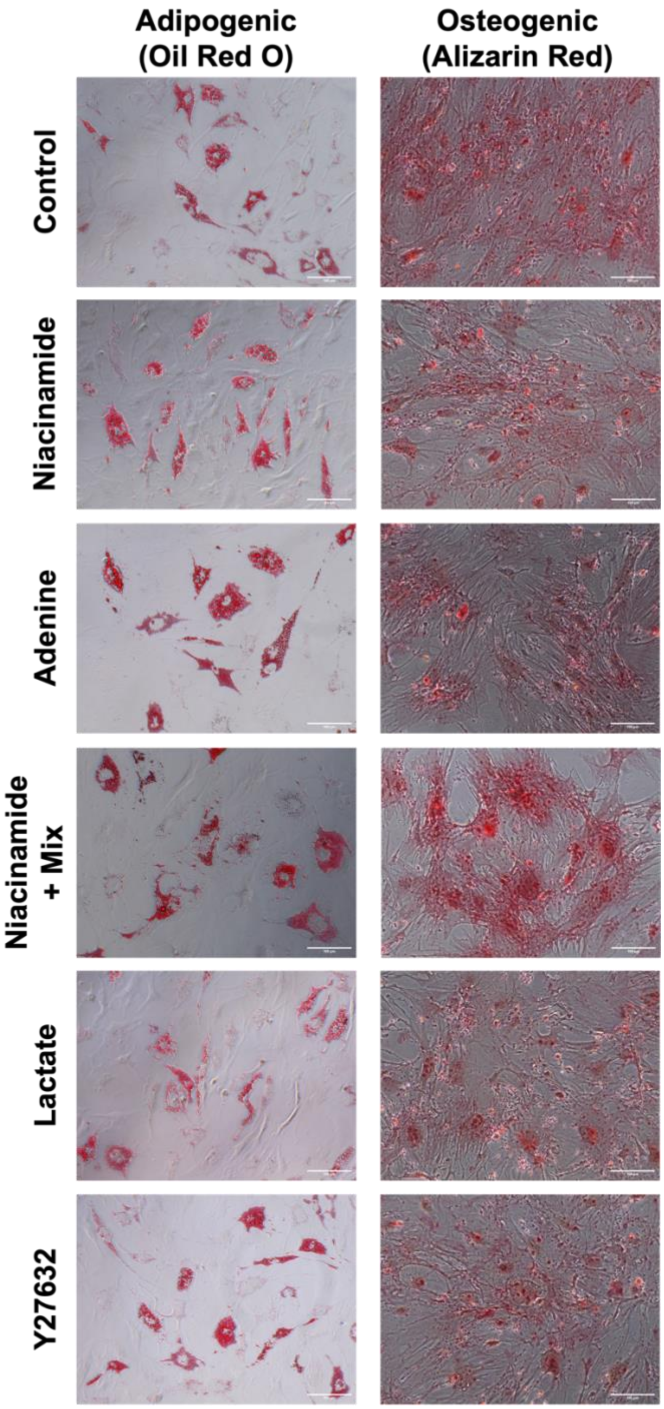

Fig. S12. Osteoblastic and adipogenic differentiation of metabolite treated MSCs. **Stro-1$^+$** MSCs were cultured for 7 days with metabolites, lactate or Y27632. Media was then replaced with adipogenic or osteoblastic differentiation media and cultured for a further 21 days. Cells were then fixed and stained with alizarin red to detect calcium deposits (Osteogenesis) or detection of lipid droplets using oli red (Adipogeneisis). Representative images shown are taken from one donor with the differentiation performed on three individual donors. Scale bars shown depict 100μm.

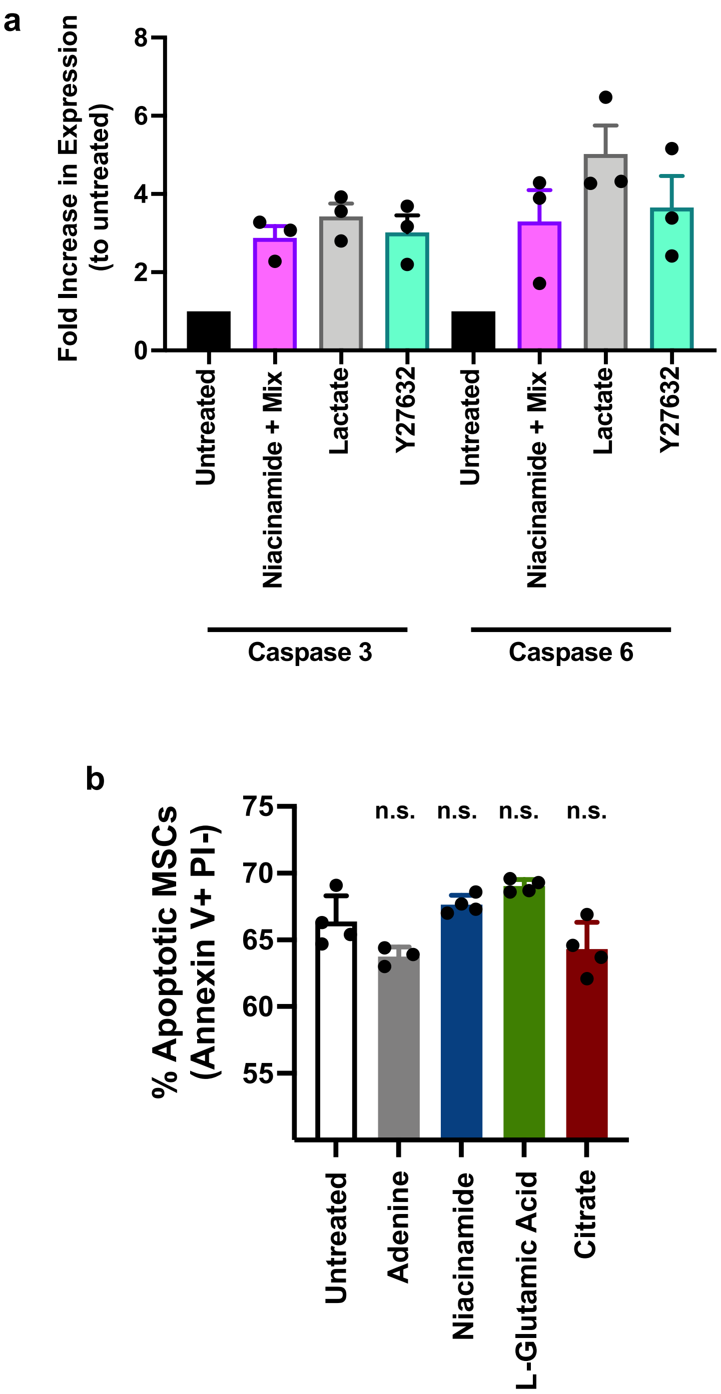

Fig. S13. Effects of metabolites on MSC apoptosis responses. (a) MSCs were cultured with a mixture of metabolites, 5mM lactate or rock inhibitor Y27632 for 7 days. Active Caspase 3 and 6 were then quantified using immunofluorescent staining (n=3 donors, mean ± SEM; Graph shows mean ± S.D., comparisons by ANOVA with Dunnett's test of multiple comparisons.). (b) MSCs were cultured in the presence of individual metabolites for 7 days and assessed for susceptibility to apoptosis when co-cultured with activated PBMCS at 1 :10 ratio using Annexin-V and PI staining by flow cytometry (mean± S.D. of n=4 replicates, representative of n=2 donors, comparisons by two way ANOVA with Dunnett's test of multiple comparisons). Source data are provided as a Source Data file.

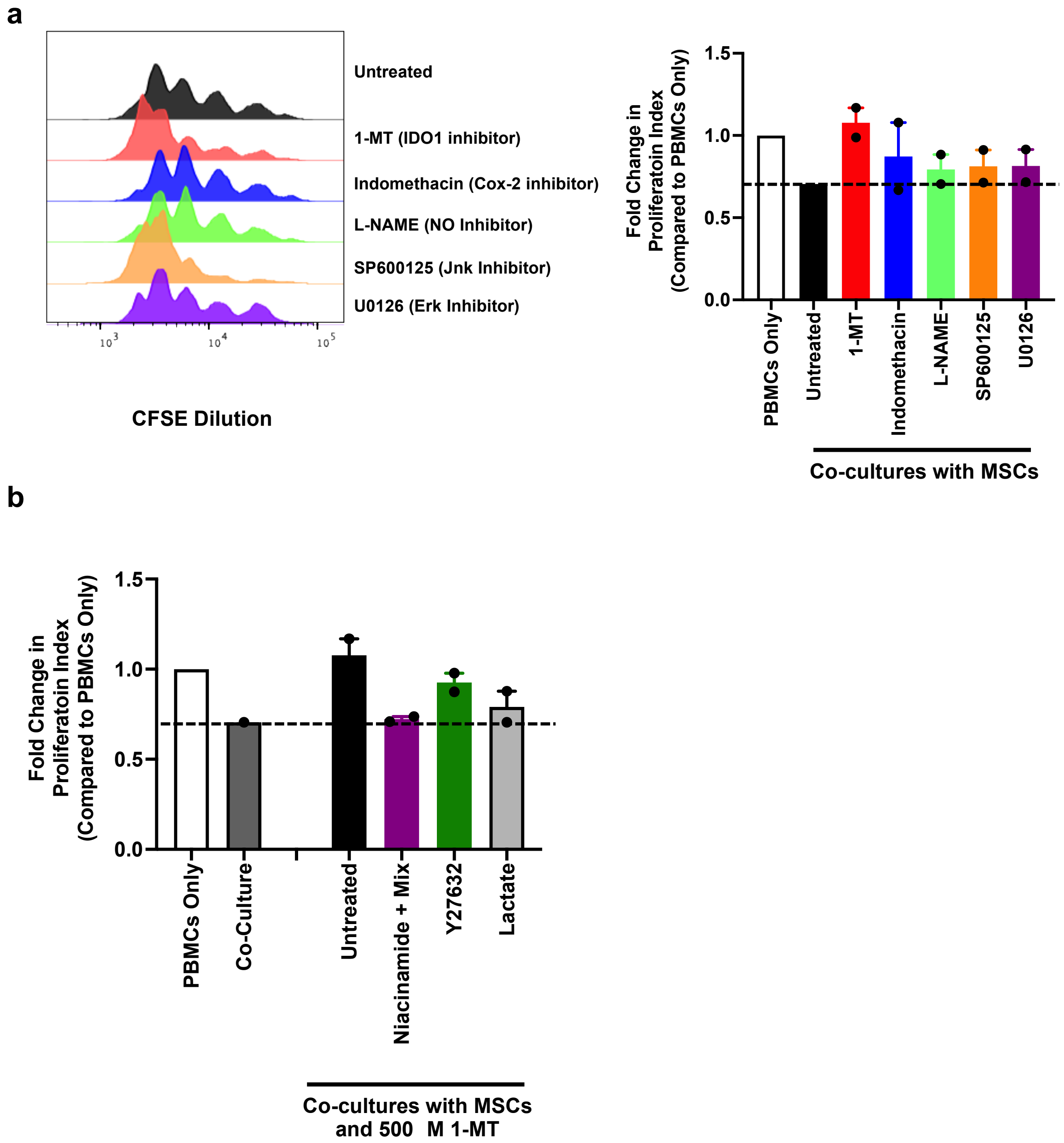

Fig. 14. Effects of pathway inhibition on Stro-1$^+$ MSC immunosuppression. (a) Stro-1+ MSCs were treated inhibitors of IDO1 activity (1-MT), Cox2 (Indomethacin), Nitric Oxide synthase (L-NAME), pan JNK inhibitor (SP600125) or ERK inhibition (U0126). Effects on the cells ability to suppress T cell proliferation were then assessed through CFSE dilution by flow cytometry. (b) Stro-1$^+$ MSCs were treated with metabolites for 7 days before co-culturing with CFSE labelled PBMCs in the presence of 1-MT for 5 days. T cell proliferation was assessed through CFSE dilution using flow cytometry, and effects on immunosuppression quantified. Graphs show mean ± SEM (n=3 co-cultures per donor) of two donors. Source data are provided as a Source Data file.

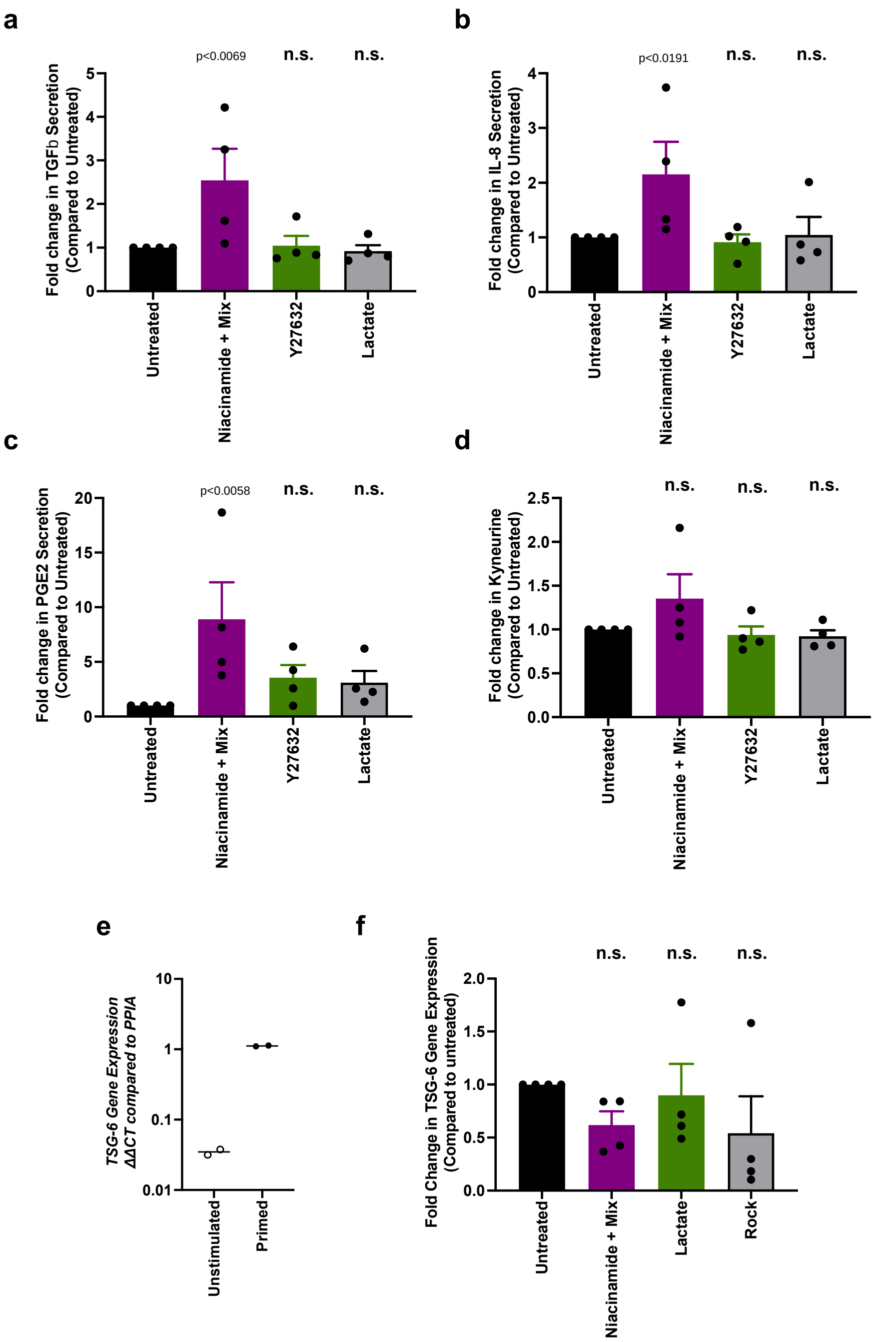

Fig. S15. Measuring immunomodulatory factors in metabolite treated Stro-1^{+} MSC secretome. Stro-1^{+} MSCs were treated with metabolites, lactate or ROCK inhibitor Y27632 for 7 days then 'primed' for 72 hours with 5 ng/ml IFNγ and 5 ng/ml TNFα. Secretion of IL-6, IL-8 and PGE2 was measured by ELISA (a-c). IDO-1 activity was measured using Ehrlich's solution to measure L-kyneurine, a breakdown product of tryptophan (d). (e) Induction of TSG-6 gene expression after IFNγ and TNFα priming by qPCR (n=2 donors). (f) TSG-6 gene expression measured after IFNγ and TNFα priming in metabolite treated Stro-1^{+} MSCs compared to untreated cells. Graphs in a-d and f show mean $\pm$ SEM of four independent donors. Comparisons by two way ANOVA with Dunnett's test of multiple comparisons). Source data are provided as a Source Data file.

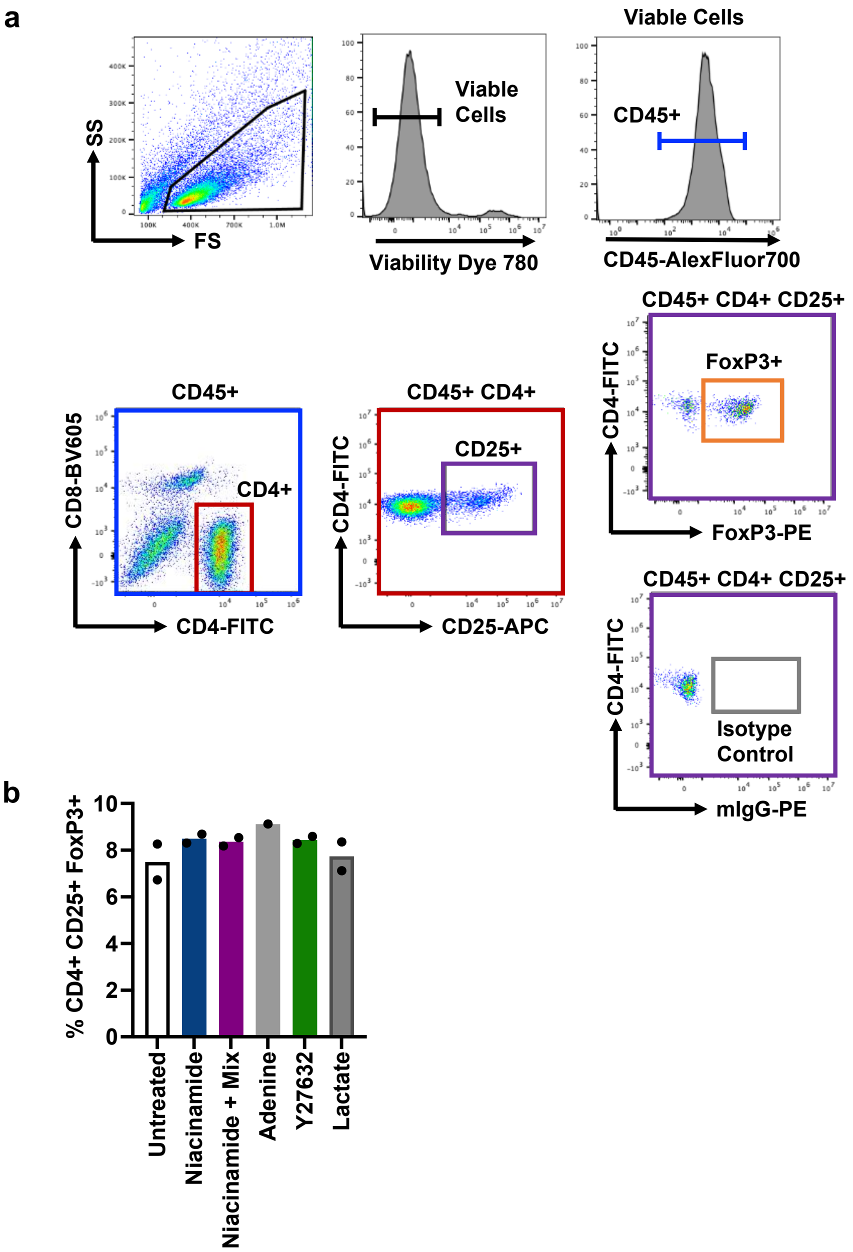

Fig S16. MSC directed increase in regulatory T cells (TRegs) during co-culture. MSCs and PBMCs were co-cultured (1:3 ratio) in the presence of 10U/ml IL-2, and the ability of treated MSCs to promote TRegs was assessed by flow cytometry. (**a**) Representative flow cytometry analysis of TRegs ($CD45^{+}$ $CD4^{+}$ $CD25^{+}$ $FoxP3^{+}$) after co-culture. (**b**) Percentage of CD4 T cells which were TRegs after co-culture for 7 days with treated MSCs (n=2 co-cultures per group). Source data are provided as a Source Data file.

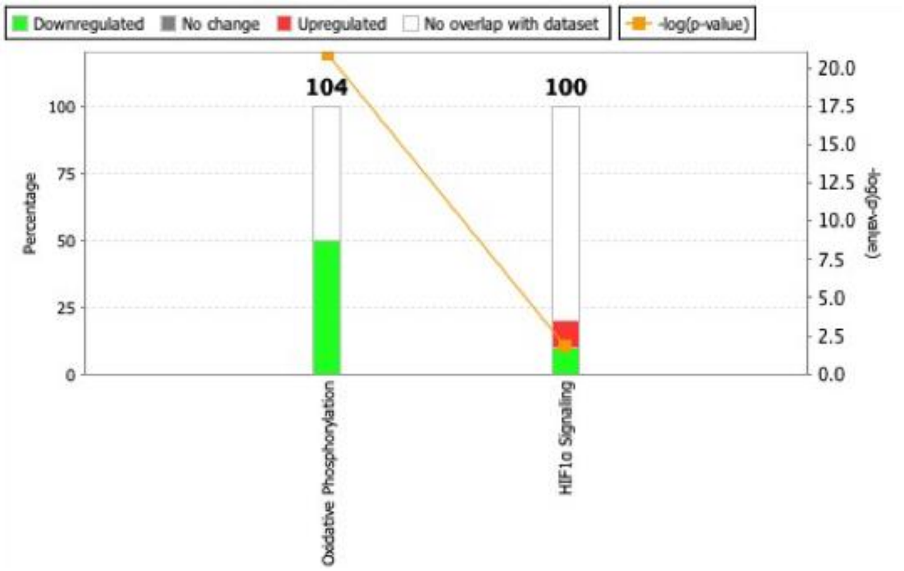

Fig. S17. RNAseq reveals changes to oxidative phosphorylation. Stro-1^{+} MSCs were cultured on SQ or flat nanotopographies for 24 hours before RNA was harvested and analysed by next generation sequencing. Changes to RNA species involved in oxidative phosphorylation and HIF-1α pathways were evaluated using Ingenuity Pathway Analysis software. n=3 material replicates per group.

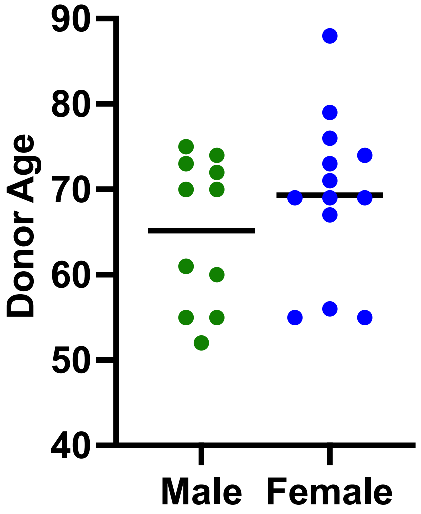

Fig. S18. Donor Stro-1$^+$ MSC diversity. This study used bone marrow derived Stro-1$^+$ MSCs from a total of 24 independent donors, with an almost equal distribution of male (n=11) and female (n=13) sources. The distribution of ages of donors was also similar between male (mean age 65.2 years old, youngest 52 and oldest 75) and female (mean age 69.3 years old, youngest 55 and oldest 88). Source data are provided as a Source Data file.

Table S1. Test liquids and their corresponding surface tensions and surface tension components used for the OWRK method.

Liquid	mN/m)	mN/m)	
		Dispersive	Polar
**Water**	72.8	29.1	43.7
**Diethylene glycol**	44.6	31.7	12.9
**Formamide**	59.0	39.4	19.6

Table S2. Reagents used for flow cytometry.

Antigen	Clone	Isotype	Fluorochrome	Source	Dilution Used
**CD4**	OKT4	mIgG2b	FITC	Biolegend	1 in 100
**CD8**	RPA-T8	mIgG1	Brilliant Violet 605	Biolegend	1 in 200
**CD25**	M-A251	mIgG1	APC	Biolegend	1 in 50
**CD29**	TS2/16	mIgG1	FITC	eBioscience	1 in 100
**CD44**	IM7	Rat IgG2b	PE-Cy7	eBioscience	1 in 500
**CD45**	2D1	mIgG1	AlexaFluor700	eBioscience	1 in 200
**CD90**	eBio5E10	mIgG1	PerCP-eFluor710	eBioscience	1 in 200
**CD106**	STA	mIgG1	PE	eBioscience	1 in 100
**CD166**	3A6	mIgG1	PerCP-eFluor710	eBioscience	1 in 50
**CD271**	REA844	REA	PE-Vio770	Miltenyi Biotech	1 in 50
**FoxP3**	259D	mIgG1	PE	Biolegend	1 in 50
**Fixable Viability Dye 780**			eFluor780	eBioscience	1 in 2000

Isotype Control	Clone	Fluorochrome	Source
Mouse IgG1k	P3.6.2.8.1	FITC	eBioscience
Mouse IgG2b	MPC-11	FITC	Biolegend
Rat IgG2bk	eB149/10H5	PE-Cy7	eBioscience
Mouse IgG2bk	MOPC-21	APC	Biolegend
Mouse IgG1k	MOPC-21	Brilliant Violet 605	Biolegend
Mouse IgG1k	MOPC-21	AlexaFluor700	Biolegend
Rat IgG2bk	eB149-10HS	PE	eBioscience
Mouse IgG1k	P3.6.2.8.1	PerCP-eFluor710	eBioscience
Mouse IgG1k	P3.6.2.8.1	PE	eBioscience
Recomb hum IgG1	REA293	PE-Vio770	Miltenyi Biotech

Table S3. Primers used for qPCR.

Gene	Forward Primer	Reverse Primer
**IDO1**	gtgtttcaccaaatccacga	ctgatagctgggggttgc
**TSG6**	tcacctacgcagaagctaaggc	tccaactctgcccttagccatc
**Housekeeping Gene**	**Forward Primer**	**Reverse Primer**
**GAPDH**	tcaaggctgagaacgggaa	tgggtggcagtgatggca
**RPL13A**	ggataagaaaccctgcgaca	gcctcgaccatcaagcac
**PPIA**	atgctggacccaacacaaat	tctttcactttgccaaacacc

Table S4. Summary of cell donors used and experimental replicates.

Figure	Experiment	Number of Donors	Biological Replicates	Technical Replicates
1c	Immunomodulation	1	1	4
1d	Immunomodulation	7	7	3-4
1e, f	Immunomodulation	1	1	4
1g	Nanoindentation	1	1	169-234
1h	Y27632 + Actin	1	1	15
1i	Y27632 + Immunomodulation	1	1	3
1j	Y27632 + Immunomodulation	3	3	4
2a, b, c	Metabolomics	3 Stro-1 donors<br>3 commercial donors	3<br>3	4<br>4
2d	Metabolomics	3	3	4
2f	JC1	4	4	3
2g	MitoTracker	4	1	1
2h	MitoSOX	4	1	1
2i	Y27632 + JC1	4	4	3-4
3b	13C-Glucose	3	3	3-4
3c	2-NBDG	4	4	3-4
3d	Lactate	3	3	1-2
4a	DNP + Immunomodulation	3	3	4
4b	qPCR	5	5	1
4c	Phenotyping	4	4	1
5a	Immunomodulation	2	2	4
5b	Metabolomics	3	3	4
5c	Metabolites and cell number	4	4	1
5d, e	Seahorse Mitochondrial Function	1	1	3
5f	Seahorse Quantification	3	3	3
5g	P-Myosin Western Blot	3	3	1
6b	Cell Stack Cell Count	2-4	2-4	1
6c	Proliferation Index	2-4	2-4	3
6e	Co-Culture Apoptosis	3	3	2-3
6f	Cell Stack Phenotype	4	4	1
S1b, c	CFSE Timecourse	2	2	4
S2	Surface Energy Measurements	1	1	10
S3a	Topography Apoptosis	2	2	3
S3c	Topography Caspase Activation	3	3	1
S5a	Nanoindentation	1	1	192-209
S5b	MSC + Activated PBMC Co-culture	1	1	4
S5c	MSC + Activated PBMC Co-culture	2	1	4
S8b, c	Super Resolution Microscopy	1	1	12
S9a	Nanoindentation	1	1	169-176
S9b	Lactate CFSE Proliferation	2	2	4
S10	Metabolomics	3	3	4
S11	Seahorse Energy Map	1	1	3
S12	Differentiation	3	1	3
S13a	Metabolite Caspase Activation	3	3	1

S13b	MSC + Activated PBMC Co-culture	1	1	4
S14a, b	Inhibitor Studies	2	1	3
S15a-d	ELISA / Kynurenine	4	1	2-3
S15e	TSG6 Priming qPCR	2	1	3
S15f	TSG6 qPCR	4	1	3
S16b	Regulatory T Cell Quantification	1	1	2
S17	Metabolomics	3	3	4
