# Supplementary material for: Nanotopography reveals metabolites that maintain the immunomodulatory phenotype of mesenchymal stromal cells
Source: Nat Commun. 2023 Feb 10;14:753. doi: 10.1038/s41467-023-36293-7 (PMC9918539; doi:10.1038/s41467-023-36293-7)
Supplement: Supplementary file 2 — Reporting Summary [file 41467_2023_36293_MOESM2_ESM.pdf]

nature portfolio

Corresponding author(s): Prof Matthew Dalby

Last updated by author(s): Jan 6, 2023

Reporting Summary

Nature Portfolio wishes to improve the reproducibility of the work that we publish. This form provides structure for consistency and transparency in reporting. For further information on Nature Portfolio policies, see our Editorial Policies and the Editorial Policy Checklist.

Statistics

For all statistical analyses, confirm that the following items are present in the figure legend, table legend, main text, or Methods section.

n/a	Confirmed	
☐	☒	The exact sample size (*n*) for each experimental group/condition, given as a discrete number and unit of measurement
☐	☒	A statement on whether measurements were taken from distinct samples or whether the same sample was measured repeatedly
☐	☒	The statistical test(s) used AND whether they are one- or two-sided<br>*Only common tests should be described solely by name; describe more complex techniques in the Methods section.*
☐	☒	A description of all covariates tested
☐	☒	A description of any assumptions or corrections, such as tests of normality and adjustment for multiple comparisons
☐	☒	A full description of the statistical parameters including central tendency (e.g. means) or other basic estimates (e.g. regression coefficient) AND variation (e.g. standard deviation) or associated estimates of uncertainty (e.g. confidence intervals)
☐	☒	For null hypothesis testing, the test statistic (e.g. *F*, *t*, *r*) with confidence intervals, effect sizes, degrees of freedom and *P* value noted<br>*Give P values as exact values whenever suitable.*
☒	☐	For Bayesian analysis, information on the choice of priors and Markov chain Monte Carlo settings
☐	☒	For hierarchical and complex designs, identification of the appropriate level for tests and full reporting of outcomes
☒	☐	Estimates of effect sizes (e.g. Cohen's *d*, Pearson's *r*), indicating how they were calculated

Our web collection on statistics for biologists contains articles on many of the points above.

Software and code

Policy information about availability of computer code

Data collection	Flow Cytometry: Attune NXT with (ThermoFisher Scientific) or Cytoflex S with v2.3 CytExpert software (Beckman Coulter) analyser; Untargeted metabolomic analysis: UltiMate 3000 Rapid Separation Liquid Chromatography with Orbitrap Liquid Chromatography Mass Spectrometry (both ThermoFisher Scientific); Heavy labelled glucose tracing: Accela 600 HPLC with Orbitrap Exactive mass spectrometer (both ThermoFisher Scientific); RNASeq: Nextseq 500 analyser (illumina); Nanoindentation: Chiaro nanoindenter (Optics 11); Realtime metabolomics: Seahorse XFe24 analyser (Agilent); Gene expression: 7500 Real Time PCR System (Applied Biosystems) or LIghtcycler 480 II (Roche); Super Resolution MIcroscopy: Elyra PS.1 microscope with 63x/1.4 oil immersion lens (Zeiss)
Data analysis	Flow Cytometry: FlowJo Software (vl0.5.3, FlowJo LLC); Untargeted metabolomic analysis: pipeline of XCMS (SCIEX) followed by MzMatch and IDEOM (both www.mzmatch.sourceforge.net), pathway analysis was performed using Ingenuity Pathway Analysis software (Qiagen), heatmaps were generated using MetaboAnalyst (www.metaboanalyst.ca); Heavy labelled glucose tracing: PeakML and Mz-Match-ISO (both www.mzmatch.sourceforge.net); RNASeq: BaseSpace Sequence Hub (Illumina); Statistics: Graphpad Prism v7; Seahorse; Wave desktop software (v2.6.1.53 Agilent; Super resolution microscopy: Zen Black software (v2.3 Zeiss)

For manuscripts utilizing custom algorithms or software that are central to the research but not yet described in published literature, software must be made available to editors and reviewers. We strongly encourage code deposition in a community repository (e.g. GitHub). See the Nature Portfolio guidelines for submitting code & software for further information.

Data

Policy information about availability of data

All manuscripts must include a data availability statement. This statement should provide the following information, where applicable:

- Accession codes, unique identifiers, or web links for publicly available datasets
- A description of any restrictions on data availability
- For clinical datasets or third party data, please ensure that the statement adheres to our policy

Source data are provided with the paper as a Source File. All data supporting the findings in this study are available within the article, can be obatined from the corresponding author or can be accessed at : http://researchdata.gla.ac.uk/973/

Human research participants

Policy information about studies involving human research participants and Sex and Gender in Research.

Reporting on sex and gender	This study uses mesenchymal stem cells isolated from consenting patients undergoing routine joint replacement. As such there was no specific age group or sex used in this study due to limited availability of donor cells. This study uses a both male and female donors, and in all experiments reported, cells from both sexes were used. The researchers have not found a discernible or significant difference in responses of male or female donor cells in any of the assays performed in this study and have not consciously biased the use of specific donors in any assay.
Population characteristics	Cells used in this study are from patients between the ages of 50-80 years old and are a mixture of male and female donors.
Recruitment	Cells were isolated from waste bone marrow taken during routine knee or hip replacement surgery. Consent was obtained from patients prior to the procedure. This approach has been validated by local ethics committees at the hospital where the procedures are performed.
Ethics oversight	Ethical approval was provided by the University of Southampton local ethics committee (NRES number: 194/99/1, LREC number 31875)

Note that full information on the approval of the study protocol must also be provided in the manuscript.

Field-specific reporting

Please select the one below that is the best fit for your research. If you are not sure, read the appropriate sections before making your selection.

☒ Life sciences ☐ Behavioural & social sciences ☐ Ecological, evolutionary & environmental sciences

For a reference copy of the document with all sections, see nature.com/documents/nr-reporting-summary-flat.pdf

Life sciences study design

All studies must disclose on these points even when the disclosure is negative.

Sample size	No statistical methods were used to predetermine sample size. They were determined based on prior experience or pilot experiments. At least n=3 technical repeats were performed per experiemntal condition in line with standards of the field
Data exclusions	No data was excluded
Replication	ll experiments were performed at least two, mostly three to four times each and each experiment gave similar results. Experimental findings were confirmed in independent experiments and found to be reliably reproducible.
Randomization	here was no intervention for randomization in this study
Blinding	Investigators were not blinded to experimental conditions given the nature of the assays performed. Metabalomic analysis was performed by a bioinformatician who had limited prior knowledge of the biological conditions, and therefore performed analysis in an unbiased fashion.

Reporting for specific materials, systems and methods

We require information from authors about some types of materials, experimental systems and methods used in many studies. Here, indicate whether each material, system or method listed is relevant to your study. If you are not sure if a list item applies to your research, read the appropriate section before selecting a response.

Materials & experimental systems

n/a	Involved in the study
☐	☒ Antibodies
☒	☐ Eukaryotic cell lines
☒	☐ Palaeontology and archaeology
☒	☐ Animals and other organisms
☒	☐ Clinical data
☒	☐ Dual use research of concern

Methods

n/a	Involved in the study
☒	☐ ChIP-seq
☐	☒ Flow cytometry
☒	☐ MRI-based neuroimaging

Antibodies

Antibodies used

All antibodies used in this study were obtained from commercial suppliers. Their details, including manufacturer and conjugate are listed below and in Table SI. All antibodies were validated by their manufacturers for use in flow cytometry on human samples that were used in this study. Antibodies were used at 1:50 dilution unless otherwise stated below.
Antibody/ Clone/ Species/ Manufacturer/ Catalogue Number/ Dilution
CD4-FITC /OKT4 /Mouse anti-human / Biolegend / 317408 / 1:100
CD8a-Brilliant Violet 605 /RPA-T8 /Mouse anti-human / Biolegend / 301039 / 1:250
CD25-APC /M-A251 / Mouse anti-human / Biolegend / 356110
CD29-FITC /TS216 / Mouse anti-human/ eBioscience / 11-0299-42
CD44-PE-Cy7 / IM7 / Rat anti-human/ eBioscience / 12-0441-82 / 1:400
CD45-AlexaFluor700 / 2D1 / Mouse anti-human / Biolegend / 368514 / 1:400
CD90-PerCP-eFluor710 / eBioSEl0 / Mouse anti-human/ eBioscience / 46-0909-42 / 1:100
CD106-PE / STA / Mouse anti-human/ eBioscience / 12-1069-42
CD166-PerCP-eFluor710 / 3A6 / Mouse anti-human/ eBioscience / 46-1668-42
CD271-PE-Vio770 / REA844 / recombinant human lgG / Miltenyi Biotech / 130-112-603
FoxP3-PE / 259D / Mouse anti-human / Biolegend / 320208
Mouse lgGl kappa isotype control FITC / P3.6.2.8.l / eBioscience / 11-4741-42
Mouse IgG2b kappa isotype control FITC / MPC-11 / Biolegend / 400309
Mouse IgG2b kappa isotype control APC / MOPC-21 / Biolegend / 400121
Rat lgG2b kappa isotype control PE/ eB149-10HS / eBioscience / 12-4031-82
Mouse lgGl kappa isotype control PerCP-eFluor710 / P3.6.2.8.l / eBioscience / 46-4714-82
Mouse lgGl kappa isotype control PE/ P3.6.2.8.1 / eBioscience / 12-4714-82
Mouse IgG1 kappa isotype control Brilliant Violet 605 / MOPC-21 / Biolegend / 400161
Mouse IgG1 kappa isotype control AlexaFluor700 / MOPC-21 / Biolegend / 400143
Recombinant Anti-TOMM20 antibody [EPR15581-39] - Mouse lgGl (ab283317) Abcam
REA lsotype Control PE-Vio770 / REA293 / Recombinant human lgG / Miltenyi Biotech / 130-104-617

Validation

All antibodies used in this study are from commercial sources and have been previously titrated by the researchers prior to use to identify their optimal concentration for the cell types being used in this study. Isotype controls were routinely used to determine non-specific background binding of the antibody to the target cell. All flow cytometry experiments used single antibody staining of representative target cells to adjust for spectral overlap between channels.

Flow Cytometry

Plots

Confirm that:

☒ The axis labels state the marker and fluorochrome used (e.g. CD4-FITC).

☒ The axis scales are clearly visible. Include numbers along axes only for bottom left plot of group (a 'group' is an analysis of identical markers).

☒ All plots are contour plots with outliers or pseudocolor plots.

☒ A numerical value for number of cells or percentage (with statistics) is provided.

Methodology

Sample preparation

Detailed methodologies covering the various sample preparation stratgies can be found in the Materials and Methods section. Mesenchymal stromal cells (MSCs): Cultured MSCs were washed with PBS and detached from their growth surface using Accutase (ThermoFisher) and single cell suspensions were typically stained on ice for 30-45mins in flow cytometry buffer (PBS + 0.5% BSA+ 0.SmM EDTA) supplemented with fluorescently labelled antibodies. For intracellular staining, fixation, permeabilisation and transcription factor staining was perfomed using the Tru-Nuclear Transcritpion Factor kit according to the manufacturer's protocol (Biolegend). was perfomred a CFSE labelled PBMCs: PBMCs were isolated from blood cones by diluting 1:1 with warm media and separating from erthryocytes and granulocytes using a Ficoll-Paque Plus density gradient according to the manufacturers instructions (GE Healthcare). Purified PBMCs were labelled with CFSE (CellTrace CFSE Proliferation kit, ThermoFisher) according to the manufacturers instructions. Mitochondrial Function: MSCs were cultured in 24-well plates and treated according to the experimental protocol. At defined times, media was removed

	and fresh warm culture media containing 2uM JC-1 (Thermofisher) was added for 20mins. Cells were then washed with warm PBS, detached using trypsin and immediately analysed by flow cytometry. Mitochondrial Mass and superoxide: MSCs were cultured with l00nM MitoTracker Green (ThermoFisher) for 30mins in serum free media before washing, detaching and immediate analysis. Mitochondrial superoxide was measured by culturing cells with SuM MitoSOX Red in HBSS for 10mins, washing, detaching and immediate analysis. Fluorescent glucose uptake: Cells were cultured in glucose free media media for 2 hours before culturing with media supplemented with 2-NBDG for 60mins. MSCs were washed, detached and immediately analysed.
Instrument	Cytometric analysis was performed using an Attune NXT flow cytometer (ThermoFisher Scientific) or a Cytoflex S analyser (Beckman Coulter)
Software	Data was collected using Attune NXT (ThermoFisher Scientific) or CytExpert (Beckman Coulter) software and analysed with FlowJo vl0.5.3 (FlowJo LLC)
Cell population abundance	In all experiments a minimum of 5000 events per sample in the live cell gate was collected and analysed to ensure statistical significance.
Gating strategy	FSC-H/SSC-H was used for gating mesenchymal stromal cells. FSC-A/SSC-A was used for gating lymphocytes. CFSE dilution in lymphocytes was identified as described in Figure S1. Changes in JC-1 staining in MSCs were analysed as described in Figure S7. Identification of regulatory T cells by FoxP3 is shown in Figure S16.

☒ Tick this box to confirm that a figure exemplifying the gating strategy is provided in the Supplementary Information.
